# Sediment core analysis using artificial intelligence

**DOI:** 10.1038/s41598-023-47546-2

**Published:** 2023-11-21

**Authors:** Andrea Di Martino, Gianluca Carlini, Gastone Castellani, Daniel Remondini, Alessandro Amorosi

**Affiliations:** 1 Department of Biological, Geological and Environmental Sciences (BiGeA), University of Bologna, Piazza di Porta San Donato 1, 40126 Bologna, Italy; 2 Department of Physics and Astronomy, University of Bologna, 40127 Bologna, Italy; 3 Department of Medical and Surgical Sciences, University of Bologna, 40138 Bologna, Italy

**Keywords:** Sedimentology, Stratigraphy

## Abstract

Subsurface stratigraphic modeling is crucial for a variety of environmental, societal, and economic challenges. However, the need for specific sedimentological skills in sediment core analysis may constitute a limitation. Methods based on Machine Learning and Deep Learning can play a central role in automatizing this time-consuming procedure. In this work, using a robust dataset of high-resolution digital images from continuous sediment cores of Holocene age that reflect a wide spectrum of continental to shallow-marine depositional environments, we outline a novel deep-learning-based approach to perform automatic semantic segmentation directly on core images, leveraging the power of convolutional neural networks. To optimize the interpretation process and maximize scientific value, we use six sedimentary facies associations as target classes in lieu of ineffective classification methods based uniquely on lithology. We propose an automated model that can rapidly characterize sediment cores, allowing immediate guidance for stratigraphic correlation and subsurface reconstructions.

## Introduction

Understanding subsurface stratigraphy is essential for a wide range of industrial and societal applications, including studies of global climate change^1,2^, reservoir characterization^3–5^, land subsidence calculations^6,7^, and engineering geology^8^. When approaching the investigation of the subsurface, by nature inaccessible to direct observation, sediment cores are the fundamental source of information. Sedimentary facies, in particular, i.e. sediment bodies or packages of strata formed in specific depositional environments, bear unique physical and mechanical properties^9^ that can be used effectively for subsurface stratigraphic modeling.

Recent studies have shown that building a detailed model of the shallow subsurface based on sedimentary facies properties can be an effective tool to: (i) assess patterns of active tectonic deformation^10^, (ii) define site response to earthquakes^11^, and (iii) predict earthquake damage risk^12^.

Sediment facies analysis is the first step in most Earth and environmental research studies; nevertheless, high-resolution facies reconstructions require specific sedimentological expertise and training, usually involving a multidisciplinary research approach^9,13,14^.

Recent advances in Artificial Intelligence research are setting new standards for many research fields, with automated methods based on Machine Learning (ML) and Deep Learning (DL) achieving state-of-the-art performance in solving complex problems. Among the principal applications of AI methods, we find Natural Language Processing (NLP)^15^, Computer Vision (CV)^16^, synthetic data generation^17^, and more. In the last few years, AI methods have been increasingly applied to Earth and environmental research^18–25^. However, the proposed approaches did not fully exploit the potential of ML and DL systems and the usage of AI in geoscience. In a recent publication, Fleming et al.^26^ pointed out the necessity for a deeper understanding of AI and automated algorithms to strengthen geosciences research policies^27–29^.

An automatic approach was recently proposed to classify Holocene sediment facies using X-ray fluorescence (XRF) scanner data^30^. This approach, however, although effective on a local scale, can hardly be exported outside the study area: XRF data rely mostly on sediment composition, which can vary greatly from site to site in the same depositional environment simply as a function of sediment dispersal.

In this context, we propose a novel approach leveraging DL to perform automatic semantic segmentation of sediment cores digital images directly acquired in the field. Semantic segmentation consists in classifying each image pixel according to a specific set of categories, and Convolutional Neural Networks (CNNs) usually achieve state-of-the-art performance^31,32^. CNNs are a particular class of networks primarily used to efficiently analyze image data. We identified six target Holocene sedimentary classes from the Po Plain and the Adriatic coastal plain of Marche, Abruzzo, and Apulia regions (Italy): Well-drained floodplain (WDF), Poorly-drained floodplain (PDF), Swamp (Sw), Peat layer (PL), Prodelta (P), and Fluvial sand (FS), deposits, with an additional background class. An expert sedimentologist manually annotated each core image, producing a final dataset of 82 non-overlapping, high-resolution digital images acquired from 32 continuous sediment cores with the associated segmentation masks. To perform the model validation, we divided the dataset into three mutually-exclusive subsets: training, validation, and test, containing 77, 11, and 12% of the data, respectively.

Our method can produce precise semantic segmentation and, thus, accurate facies interpretation, achieving high scores for the most used segmentation metrics. Our approach can drastically reduce the time and effort required to analyze core surveys; it can perform real-time predictions of high-resolution images on a regular computer and could be extended to mobile devices, making it suitable for on-field usage. This method does not need expensive data acquisition techniques or pre-processing, since it relies on images acquired with common digital cameras. Moreover, this approach is not necessarily limited to Holocene successions and could be adapted to different geologic conditions.

One of the major concerns with Deep Learning methods is the limited interpretability of model predictions. This is the reason why they are commonly called black-box methods^33–35^. In an attempt to better understand the results produced by our method, we visualize the regions of higher segmentation error and model prediction confidence, enhancing the results interpretability.

## Results

### Segmentation performance and visual evaluation

A deep CNN was trained to automatically produce the semantic segmentation masks of digital images acquired from continuous sedimentary cores. The images included six target classes corresponding to the observed sedimentary facies and a seventh background class. The model performance was evaluated on validation and test sets consisting of 11 and 12% of the total available data, respectively. We measured several standard segmentation metrics: the mean Intersection over Union (IoU), the F1-score, and the balanced accuracy. The results obtained for the validation and test data are shown in Table 1. The performance achieved on the validation dataset tends to be positively biased due to the model being fine-tuned on it, whereas the test performances are more rigorous in evaluating the capabilities of the model. The scores obtained with both datasets show no remarkable differences.

**Table 1:** Model performance obtained on validation and test data, in terms of mean Intersection over Union, F1-score, and balanced accuracy.

	Mean IoU	F1-score	Balanced accuracy
Validation set	0.884	0.936	0.905
Test set	0.853	0.916	0.861

**Figure 1:**
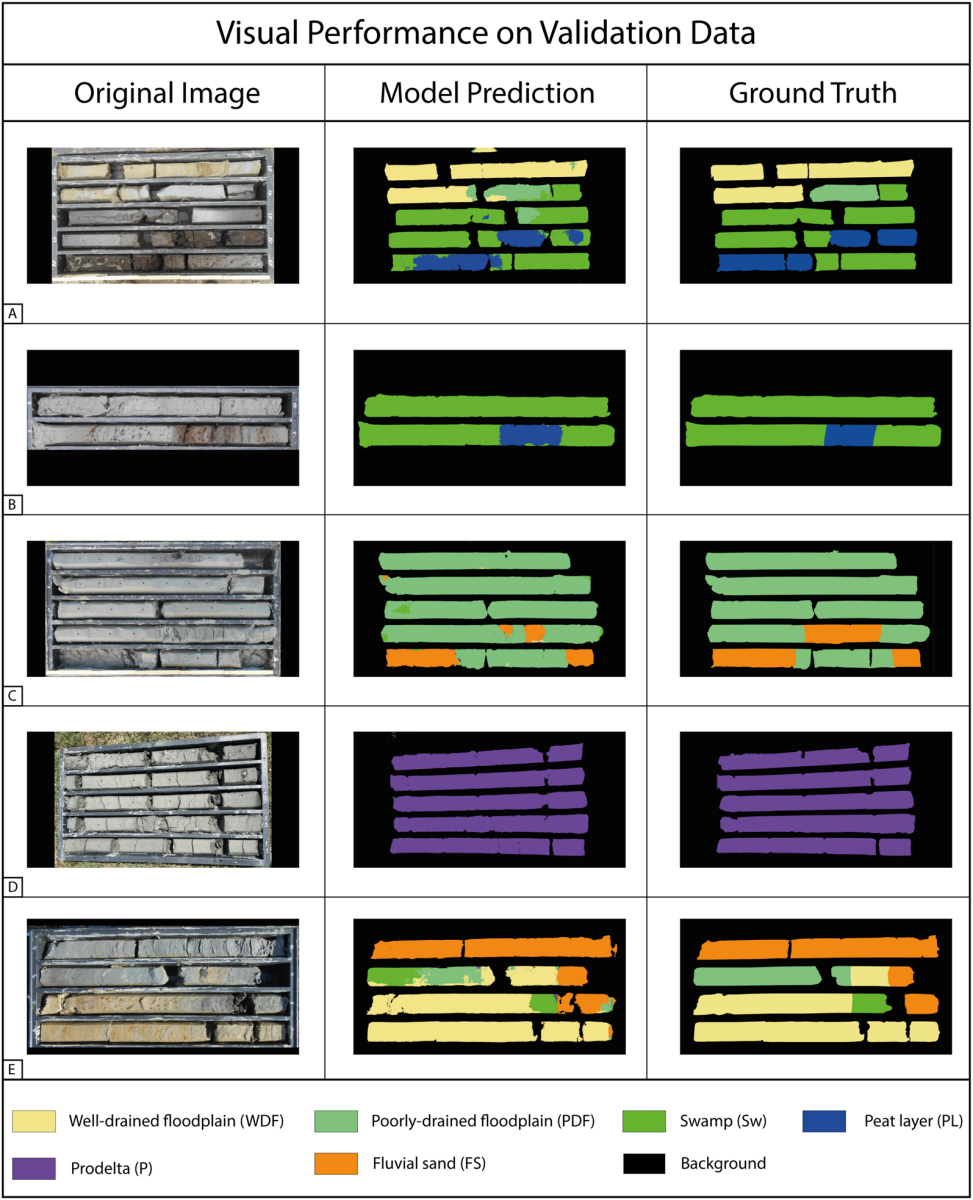
Visual performance of the model on five representative images of the validation dataset. The original full-resolution digital images, the model-produced segmentation masks, and the corresponding ground truths are shown in the left, central, and right columns, respectively.

**Figure 2:**
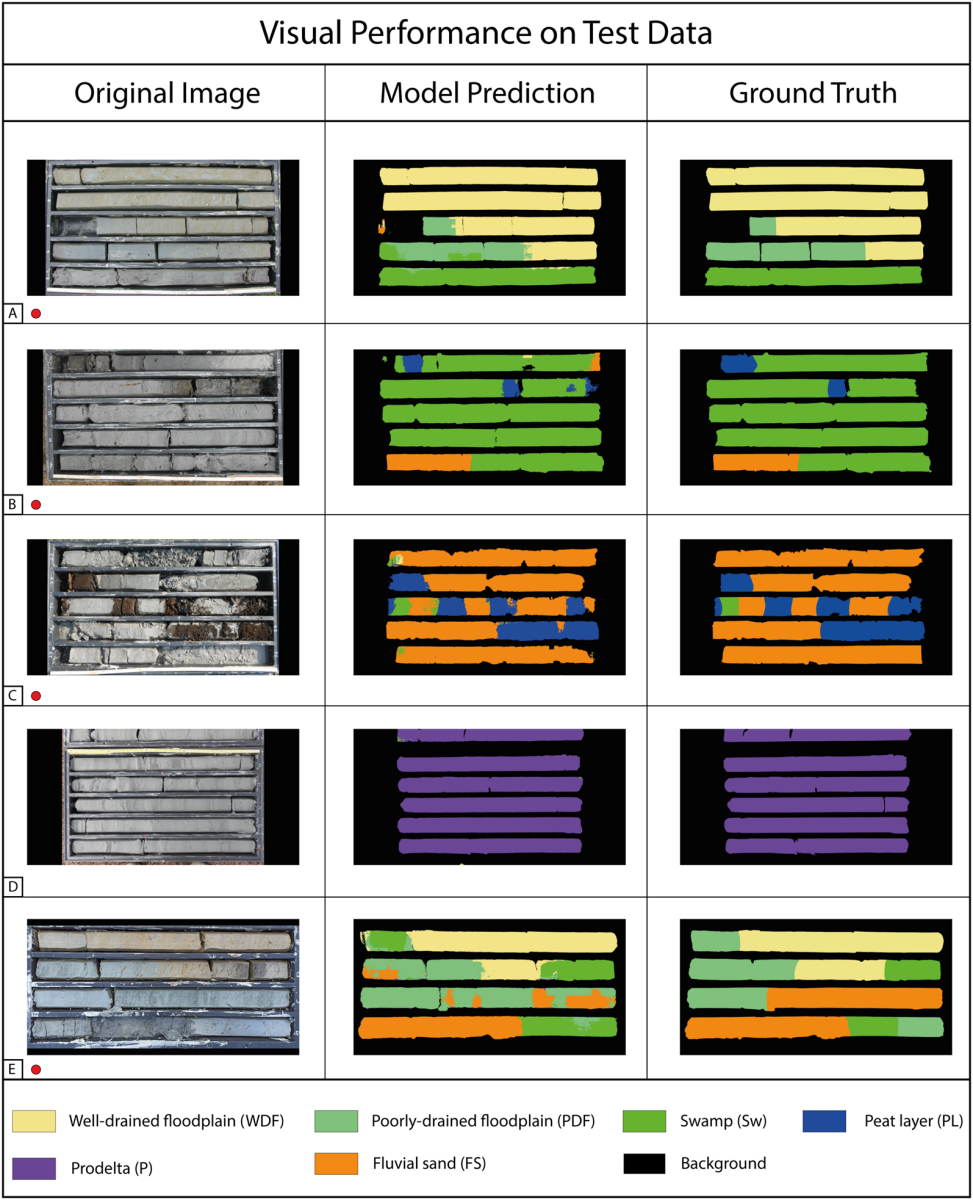
Visual performance of the model on five representative images of the test dataset. The original full-resolution digital images, the model produced segmentation masks, and the corresponding ground truths are shown in the left, central, and right columns, respectively. The red dots mark the images coming from the external set of sediment cores.

**Figure 3:**
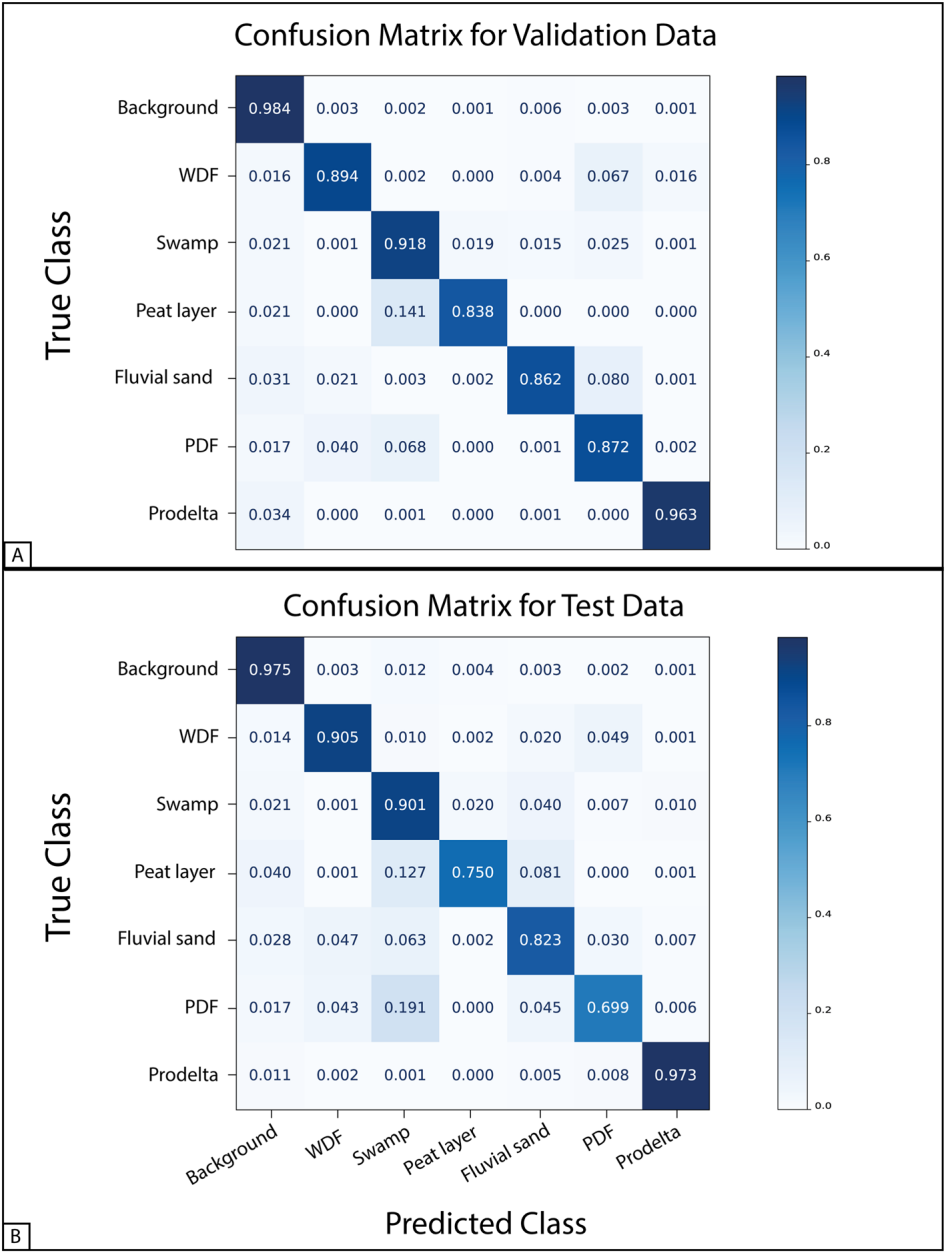
Confusion matrices for validation data (**A**) and test data (**B**). Each row of the matrices represents the instances in a ground truth class, while each column represents the class instances predicted by the model. The values were normalized with respect to the number of ground truth instances for each class. A colormap visually highlights the higher values with darker shades of blue.

For a visual evaluation of the model performance, we produced the semantic segmentation mask of five full-resolution images from both the validation and test sets and compared them to the ground truths produced by the expert sedimentologist (Figs. 1 and 2). The detailed quantitative presentation of the results and errors is covered in Section “confusion matrix and model misclassifications” and shown Fig. 3. The five images were chosen as the most representative for the two datasets, showing all the target sedimentary facies. The visual performance on validation data shows high correlation between the model predictions and the ground truths. Figure 1A is one of the most complex images in the whole dataset, containing four target classes: Well-drained floodplain (WDF), Poorly-drained floodplain (PDF), Swamp (Sw), and Peat layer (PL). The model prediction accurately reproduces the sedimentologist segmentation mask, correctly classifying most sedimentary facies. Minor errors are present, mainly localized at facies transitions (WDF–PDF, PDF-Sw, and Sw-PL). The transition between Swamp and Peat layer is also visible in Fig. 1B and well classified by the model. In Fig. 1C, the model misclassified a portion of the Fluvial sand (FS) stratigraphic interval, while classifying the PDF correctly. Figure 1D shows the high capability of the model in classifying the Prodelta (P) target class, whose identification commonly requires specific sedimentological training, with an impressive overlap between the model prediction and the reference segmentation. The model performs well in Fluvial sand classification in Fig. 1E, while only minor classification errors are present for Sw and PDF.

There are no noticeable visual performance differences between the validation and test images. Furthermore, four out of the five images shown in Fig. 2 come from a set of sediment cores for which no images were present in the training set (red dots in Fig. 2). The usage of this set of images was intended to simulate the model application to external data acquired in the field, to validate its generalization capabilities. In Fig. 2A, WDF, PDF, and Sw are correctly classified, with satisfactory predictions also near facies transitions. Negligible errors are present, with a minor misclassification of PDF to Sw; Fig. 2B, C, D) shows an almost perfect model prediction for the four involved classes. The model confirms its robust prediction capabilities of sub-features in the sedimentary record, represented by PL. Consistent with the validation result, the Prodelta class is very well classified also in the test dataset. In Fig. 2E, WDF is correctly classified, while minor errors occur on Sw and PDF classification. However, in this case the model struggles to reproduce the sedimentologists sand classification.

### Confusion Matrix and model misclassifications

For a deeper quantitative evaluation of the model performance, the confusion matrices for the validation and test sets were calculated (Fig. 3A–B). The confusion matrix is a table layout in which each row represents the instances in a ground truth class, while each column represents the class instances predicted by the model. In a semantic segmentation context, the instances are the pixels associated with each class. This matrix shows which classes have been correctly classified and which were confused with other classes during the model test. It is a standard estimator used in machine learning and statistics, since it provides more information about model performance than the standard metrics^36^. Most of the standard evaluation metrics can be derived from the confusion matrix, so it can be considered the most comprehensive method for performance evaluation in classification problems^37–40^. However, the confusion matrix becomes more complex to read with the growing number of classes.

We normalized the confusion matrix with respect to the number of true instance classes, i.e., to the rows of the matrix. It follows that the resulting matrix values are between zero and one, with one representing a perfect classification; a colormap was used for a more intuitive visual evaluation.

The confusion matrix for the validation data (Fig. 3A) shows good classification performance for all the classes. Excluding the background, the class with the highest classification accuracy is the Prodelta (0.963), followed by: Swamp (0.918), Well-drained floodplain (0.894), Poorly-drained floodplain (0.872), Fluvial sand (0.862), and Peat layer (0.838). The most significative classification errors occur for the PL, being misclassified as Sw (0.141), FS misclassified as PDF (0.080), PDF confused as Sw (0.068), and WDF confused as PDF (0.067).

The highest classification accuracy for the test data, excluding the background, is achieved again for Prodelta (0.973), followed by: Well-drained floodplain (0.905), Swamp (0.901), Fluvial sand (0.823), Peat layer (0.750), and Poorly-drained floodplain (0.699). On average, the classification accuracies are slightly lower than the validation ones, in agreement with the metrics reported in Table 1. In this case, the most significative classification errors occur for the PDF being misclassified as Sw (0.191), followed by: PL misclassified as Sw (0.127) and FS (0.081), and FS confused as Sw (0.063).

The results reported in Table 1, the visual performance shown in Figs. 1 and 2, in combination with the confusion matrices of Fig. 3, show a robust classification result for all the target classes with only minor errors.

**Figure 4:**
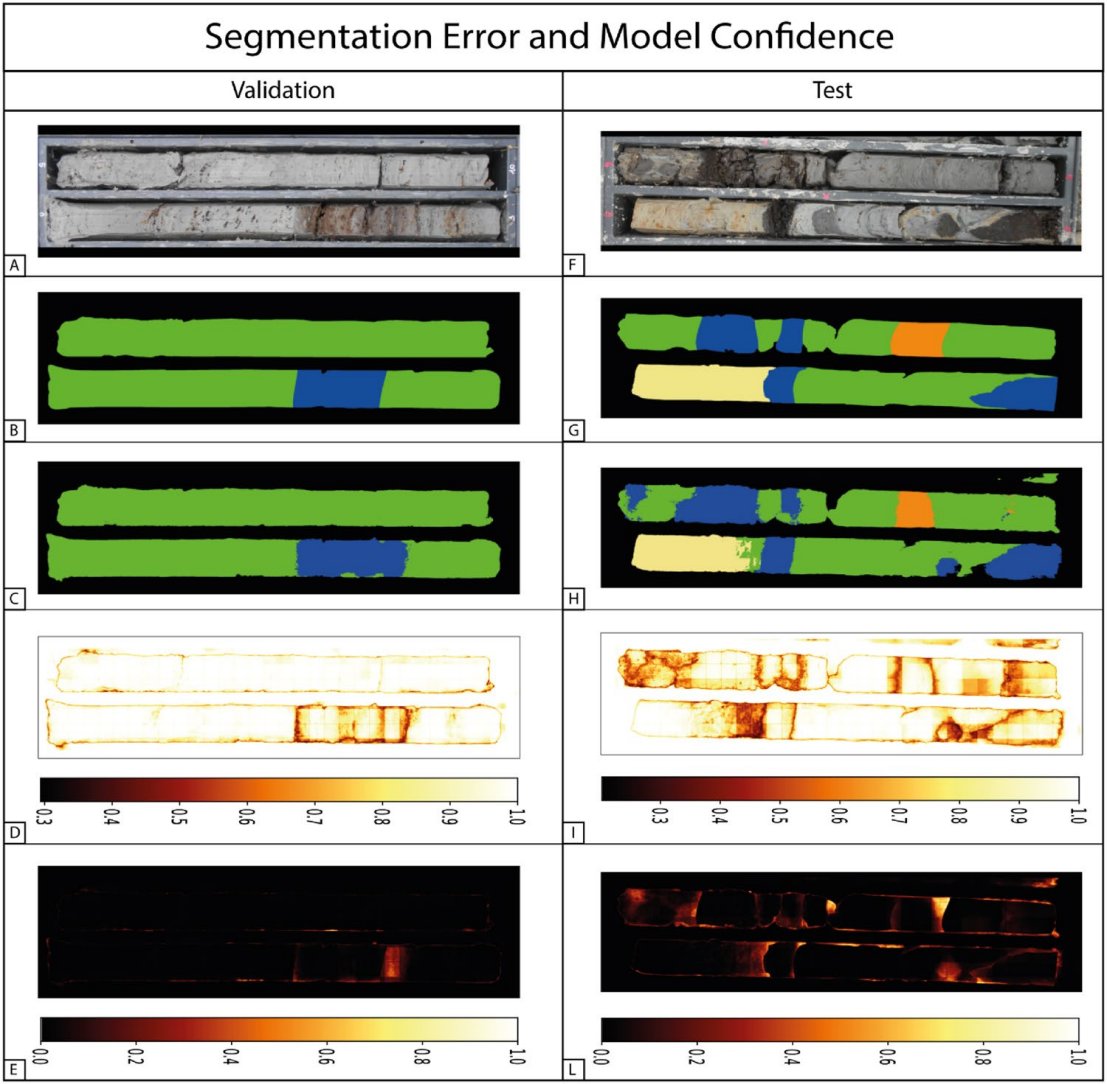
Segmentation error and model confidence. Core images (**A**–**F**), ground truth segmentation masks (**B**–**G**), model predictions (**C**–**H**), model confidence (**D**–**I**), and segmentation error (**E**–**L**) are shown for two validation and test representative cores. The model confidence represents the prediction probability associated with the predicted class. The segmentation error is the normalized categorical cross-entropy calculated between the prediction and the ground truth.

To better understand the limitations of the proposed CNN and the possible sources of error, we produced the error maps between model predictions and ground truths, along with the model prediction confidence (Fig. 4), for two representative cores from the validation and test datasets. The sediment core, ground truth, segmentation mask, and prediction are shown in Fig. 4A, B, C, F, G, H, respectively. In Fig. 4D–I, the model confidence is presented, with darker regions representing areas of lower confidence. Figure 4E–L reports the error between the prediction and the ground truth.

The validation core (Fig. 4A) shows a prominent Peat layer as a sub-feature in the Swamp deposit, with no well-defined boundaries corresponding with the minimum confidence values, as shown in Fig. 4D. The model error is restricted to a smaller portion of the image, while no major errors are noticeable from other areas of the sedimentary core.

The test image (Fig. 4F) shows a possible real-case scenario with a poorly preserved core that was damaged during drilling operations. The high number of transition boundaries makes the prediction challenging; in Fig. 4I, a generally lower model confidence is visible, with minima located in correspondence of facies transitions and on the damaged parts of the core.

## Discussion

A powerful method leveraging DL and CNNs is proposed to produce accurate sedimentary facies interpretations starting from standard digital images. It constitutes a fast, precise, and easy-to-deploy tool that could largely improve subsurface stratigraphic modeling, making subsurface facies analysis accessible to a wider range of scientists and professionals.

Using a convolutional neural network, we aimed to mimic the sedimentologist approach in facies classification; the model makes its prediction pixel-wise, but the decision process also considers local and global aspects of the image, such as the textural characteristics, color, and accessory materials, e.g., carbonate concretions, shells, and wood fragments^31,41^. The heterogeneity of the images used in the work and the data augmentation performed should make the model robust to different image lighting, contrast, orientation or quality.

The model performance obtained on the validation and test datasets shows robust generalization capabilities, with a strong agreement between the predicted classes and the ground truths identified by the sedimentologist. Furthermore, the scores obtained by the model in terms of mean IoU, F1-score, and balanced accuracy are notably high, considering the complexity of a multi-class semantic segmentation task. The numerical results are visually confirmed by the segmentation mask shown in Figs. 1, 2.

Swamp and Poorly-drained floodplain facies can have similar characteristics in terms of color and texture, thus can hardly be distinguished by visual inspection only. In such cases, the fossil content and geotechnical properties are fundamental tools for high-resolution facies analysis^9,42–44^. Our model generally performs well in discriminating these highly similar facies using visual information only, with minor errors. However, misclassification of Sw to PDF and vice-versa can hardly be considered real errors, because the distinction between these two classes is subtle and attributions could vary as a function of the sedimentologist expertise. Another typical misclassification error is due to the abundance of sand layers as sub-features within a clay deposit, such as in the case of WDF and PDF. These layers could be considered minor attributes by the sedimentologist, and thus ignored, or emphasized and interpreted as a fluvial facies. A further source of error is transition between sedimentary facies, which makes precise identification of their boundary challenging. The definition of a sharp boundary is a consequence and limitation of the semantic segmentation tasks; in some cases, however, the transition between two facies is not an abrupt change and should be considered more properly as a zone of transition. In this case, the model is not capable of fully reproducing the sedimentologist interpretation, as it may become highly subjective.

Given the black-box nature of neural networks, the information arising from the confidence and error maps can be used to highlight the criticisms of the proposed segmentation task. From Fig. 4, we acknowledge that overall the model correctly reproduces the sedimentologist interpretation.

For the validation core, the model error is restricted to a smaller portion of the image, and the overall stratigraphic interpretation does not change; moreover, the misclassified region corresponds to a facies transition, for which the identification of a sharp boundary is not straightforward, even for a sedimentologist. The test image shows a complex case in which the core was damaged during the drilling process. Also in this case, segmentation errors do not significantly impact the global stratigraphic interpretation. The damaged portions of the core are also well classified, though with a lower model confidence.

In summary, the sources of error can be grouped into three main categories: (i) visual overlap of sedimentary facies, (ii) occurrence of transitional facies boundaries, and (iii) subjectivity in sedimentologist interpretations.

There is growing interest in the application of AI methods to environmental and Earth sciences^18,19,25,45^. In this paper, we outline a novel approach to subsurface stratigraphy, performing semantic segmentation of Holocene sedimentary facies with convolutional neural networks. Previous subsurface studies have relied upon standard machine learning techniques, and for this reason they have been limited to simple data structures^20–22,30,46^. Our method leverages the power of deep learning models to produce accurate stratigraphic interpretations starting from digital images. This makes our approach easier to use and deploy in a wide range of geological applications.

During the last decades, a virtually continuous stratigraphic record of Holocene deposits in the Po Plain and beneath several coastal plains of Italy was acquired through core drilling. Robust high-resolution sequence-stratigraphic models that rely on information extracted from cores^47^ demonstrated that the stratigraphic architecture of Holocene successions worldwide exhibits striking similarities in terms of sediment facies distribution. Sediment core analysis based on semantic segmentation of continuous core images, thus, represents a highly reproducible technique that is likely to be exported successfully to other coeval stratigraphic successions, constituting a reference framework for future CNN-based subsurface analysis. The research approach presented in this paper is naturally suited for in-situ analysis and could substantially reduce the time and effort needed for detailed sediment facies interpretation, making it a valuable tool for large-scale exploration and for a broad range of industrial applications. The future integration of imaging data with other data sources, such as geotechnical and compositional data, and their incorporation in an automated method based on machine learning can make a substantial contribution to the progress of geological research below the ground surface.

## Materials and methods

### Data acquisition and pre-processing

**Figure 5:**
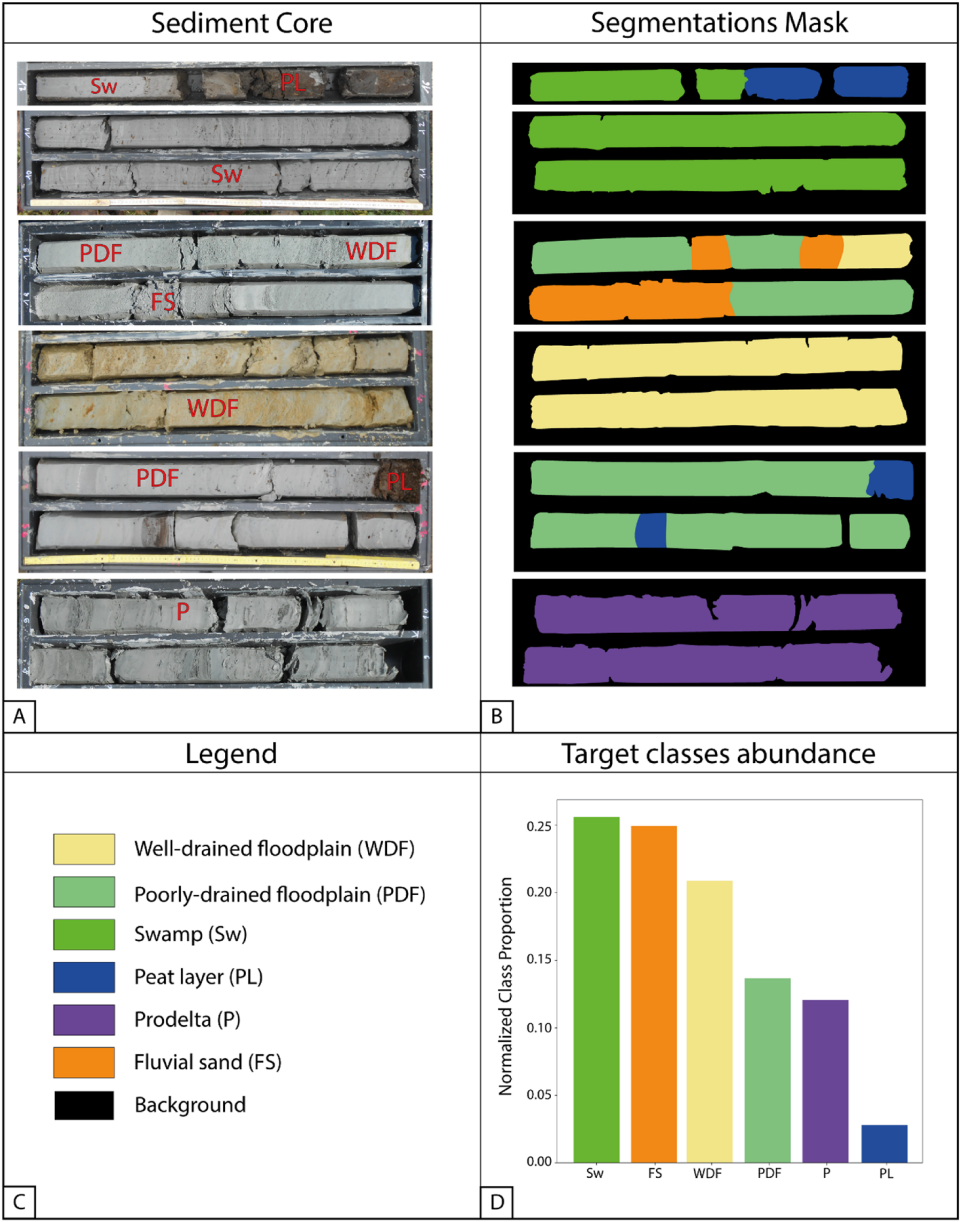
(**A**) Examples of digital images of continuous sediment cores with associated segmentation masks (**B**). (**C**) Target classes and background colors. (**D**) Relative target classes abundances.

The dataset^48^ used for this study consists of 82 digital images from 31 selected Holocene cored sedimentary successions of Italy (Po Plain and Adriatic coastal plains of Marche, Abruzzo, and Apulia regions), between December 2016 and July 2021 (Fig. 5A). Given the remarkable length (30–50 m) of sediment cores, a series of non-overlapping digital images were acquired every 5 m of recovered sediment, covering the whole core length. Digital images were obtained directly in the field using different devices, such as compact cameras and smartphones, with a broad range of resolutions, ranging from 1369 × 803 to 4605 × 2717 pixels. To make our method as general as possible, we did not enforce a strict image acquisition procedure in terms of camera settings and environmental conditions. Each image was resized to 3074 × 1538 pixels to obtain a homogeneous resolution. Whenever the aspect ratio of the target resolution was different from the aspect ratio of the image, this latter was padded with zero-valued pixels to maintain its original aspect ratio, i.e., to resize the image without distortions. No other pre-processing steps were carried out on the original images.

Using pre-existent, high-resolution stratigraphic reconstructions as a framework for facies interpretations^10,13,14^, we produced the ground truth segmentation mask for every image (Fig. 5B), manually classifying the sediment core into seven classes: six target classes corresponding to the observed Holocene sedimentary facies, and a seventh background class. A segmentation mask is an image mapping each identified class to a corresponding unique RGB value. RGB masks are suited for visualization, but are not directly usable to train a segmentation model; thus, we quantized the original RGB masks, mapping each RGB triplet to a corresponding unique scalar value. There is a slight variation in the relative proportion of the six target classes, particularly for the Peat layer class, which is only locally observed, as it occurs at distinct stratigraphic intervals (Fig. 5).

The seven classes are as follows:

(i) Well-drained floodplain deposits (WDF), typical of subaerially exposed environments, consist of stiff, varicolored light grey to green silt and clay, with yellowish and orange mottles due to Fe oxides. White carbonate concretions and other pedogenic features are common.
(ii) Poorly-drained floodplain deposits (PDF), characteristic of flood basins with fluctuating groundwater table, consist predominantly of soft, grey clay and silt, with scattered vegetal remains and a lack of body fossils.
(iii) Swamp deposits (Sw), typical of waterlogged environments, are dominated by grey to dark‐grey clay, with abundant vegetal remains and wood fragments concentrated in discrete horizons or scattered along core sections.
(iv) Peat layers (PL) represent sub-features of swamp deposits, characterized by dark grey to black colors. They consist of wood fragments with subordinate clayey material and are typically organic-matter-rich.
(v) Prodelta deposits (P) consist of homogenous, light grey clay formed at fluvial mouths, with common silt and sand intercalations, interpreted to represent flood layers. Plant debris and other organic matter are locally observed, whereas salt-water mollusks are common.
(vi) Fluvial sand (FS) includes a wide range of grain size fractions, from silty sand to very coarse sand, formed in fluvial/distributary channels or in adjacent areas (levees and crevasse splays).
(vii) Background, corresponding to the grey box containing the sediment core.

The dataset was then divided into three, non-overlapping portions; 63 digital images were used for training, 9 for validations, and 10 for testing, corresponding to 76.83%, 10.97%, and 12.20% of the total, respectively. The data were stratified, so that each class was equally represented in every subset. To further validate the generalization capabilities of the model, one image in the validation dataset and six images in the test dataset were taken from sediment cores that were not present in the training dataset.

Due to computational limitations, full-resolution images could not be used for model training. Thus, following data subdivision, the images and corresponding masks were subdivided into (i) 1609 non-overlapping patches of resolution 384 × 384 pixels for model training; (ii) 250 patches for model validation; and (iii) 265 patches for model testing. Patch subdivision is also useful for increasing the number of available samples. The padding with zero-valued pixels during the resize operation could lead to the generation of patches with mostly zero-valued pixels at image borders. For this reason, patches with less than 5% non-zero pixels were automatically excluded from the data during the patch subdivision process.

### Segmentation model

The model used for the image segmentation was a U-Net^49^ with an EfficientNetB3 backbone^50^, with weights pre-trained on ImageNet^51^. We chose the EfficientNetB3 as the backbone for the segmentation model because EfficientNets can achieve better performance than other popular model architectures^52^, such as ResNets^53^, while having a smaller number of parameters. Furthermore, to avoid overfitting, considering the limited number of training samples, we decided to employ a network with a relatively small number of parameters. Moreover, a lightweight model may also be used for real-time predictions without requiring a dedicated, powerful hardware.

The model was trained for 100 epochs using Adam^54^ as optimizer, with a starting learning rate of 10^–4^ and a polynomial learning rate decay schedule. Data augmentation was used to improve the generalization capabilities of the model. The transformations used for data augmentation were: random rotation with a 360-degree range, random brightness variation, and random contrast variation. The loss function used for the model training was the categorical cross-entropy. During training, we monitored the mean Intersection over Union (IoU) as a measure of the model performance, and we saved the model weights achieving the highest mean IoU on the validation data. The IoU is a typical metric used in segmentation tasks; it measures the overlap between the predicted mask and the ground truth^55^. The IoU is zero when there is no overlap between the prediction and the ground truth, while it is equal to one for a perfect overlap.

We used the trained model to predict the image patches in the validation and test sets, and we computed the mean IoU, the F1-score, the balanced accuracy, and the confusion matrix to measure the prediction performance^56,57^. The convolutional neural network was built using the Tensorflow^58^ python library, while the metrics and confusion matrix were calculated with the Scikit-learn^59^ python library. In detail, the IoU, F1-score and accuracy are defined as follows:

$$ F1 = \frac{{2 \times {\text{Precision}} \times {\text{Recall}}}}{{{\text{Precision}} + {\text{Recall}}}} $$

where

$$ {\text{Precision}} = \frac{TP}{{TP + FP}} $$

$$ {\text{Recall}} = \frac{TP}{{TP + FN}} $$

where TP is True Positive, FP is False positive, and FN is False Negative

$$ {\text{IoU}} = \frac{TP}{{TP + FP + FN}} $$

$$ {\text{Accuracy}} = \frac{TP + TN}{{TP + TN + FP + FN}} $$

where TN is True Negatives.

All the metrics were weighted with respect to the number of true instances for each class to take into account the classes unbalancing.

To predict a whole image, a sliding-window approach is used. A kernel of *patch-size* slides through the image with a stride of 96 pixels, corresponding to one-fourth of the patch dimension. The image portion identified by the kernel is given to the model to produce the prediction; then, the predicted regions are re-arranged to form the complete predicted image. With a stride smaller than the kernel dimension, the resulting predictions will overlap, with an overlapping portion depending on the stride size. The overlapping predictions are then averaged to produce the final predicted image, achieving a smoother result.

We produced visual maps for a thorough estimation of model errors and prediction confidence; we define as model prediction confidence the probability associated with the predicted class. The model confidence cannot be directly related to a measure of prediction uncertainty, since it could predict the wrong class, while being highly confident of its prediction. The error between the prediction and the ground truth is calculated as the normalized categorical cross-entropy, defined, for a single data point, as:

$$ H\left( {y,\hat{y}} \right) = - \mathop \sum \limits_{i = 1}^{C} y_{i} \log \left( {\hat{y}_{i} } \right) $$

where $\mathrm{C}$ is the number of classes, $y$ is the true probability distribution, and $\widehat{y}$ is the predicted probability distribution from the model.

## Data Availability

All the data used in this study can be found at https://doi.org/10.6092/unibo/amsacta/7308 preserved in the repository AMSacta hosted by the University of Bologna and licensed under Creative Commons 4.0.

## Code availability

All the code developed for this study can be found at GitHub—GianlucaCarlini/CoreNet: Semantic segmentation of continuous cores with CNNs.
